# *ADIPOQ* and *IL6* variants are associated with a pro-inflammatory status in obeses with cardiometabolic dysfunction

**DOI:** 10.1186/s13098-015-0027-2

**Published:** 2015-04-11

**Authors:** Raquel de Oliveira, Tamiris Invencioni Moraes, Alvaro Cerda, Mario Hiroyuki Hirata, Cristina Moreno Fajardo, Marcela Correia Sousa, Egidio Lima Dorea, Márcia Martins Silveira Bernik, Rosario Dominguez Crespo Hirata

**Affiliations:** School of Pharmaceutical Sciences, University Sao Paulo, Av. Prof. Lineu Prestes, 580, 05508-900, Sao Paulo, SP Brazil; Center of Molecular Biology and Pharmacogenetics, BIOREN-CEGIN, Universidad de La Frontera, Temuco, Chile; University Hospital, University of Sao Paulo, Sao Paulo, Brazil

**Keywords:** Obesity, Inflammation, Adiponectin, Interleukin-6, Gene polymorphism

## Abstract

**Background:**

Polymorphisms in genes encoding adiponectin (*ADIPOQ*) and interleukin-6 (*IL6*) have been associated with adiposity and obese-related phenotypes. This study investigated the relationship of *ADIPOQ* and *IL6* gene polymorphisms with pro-inflammatory and cardiometabolic markers in obese patients.

**Methods:**

Anthropometric and body composition parameters were measured in 249 Brazilian subjects (30 to 68 yr). Metabolic and inflammatory markers and adipokines were analyzed in blood samples. *ADIPOQ* rs2241766 (45 T > G) and *IL6* rs1800795 (−174G > C) polymorphisms were analyzed by real-time PCR and PCR-RFLP, respectively.

**Results:**

Type 2 diabetes, hypertension, dyslipidemia and increased values of waist circumference, body fat, leptin, fibrinogen, IL-1β, hsCRP and TNFα were related to obesity (p < 0.05). Multiple linear regression analysis showed a positive correlation between BMI and waist circumference, body fat, leptin, fibrinogen, PAI-1, IL-1β, hsCRP and TNFα values (p < 0.001) but not with adiponectin. Obese group had altered metabolic status, resistance to leptin and insulin, and atherogenic and pro-inflammatory profiles. *ADIPOQ* and *IL6* variants were not directely related to obesity, leptin resistance or alterations in cardiometabolic markers. Individuals carrying *ADIPOQ* 45G allele (TG + GG genotype) had higher IL-6, IL-1β and TNFα levels than TT genotype carriers (p < 0.05). *IL6* -174GG genotype was associated with increased IL-1β levels (p = 0.033).

**Conclusion:**

Obesity is associated with leptin resistance, cardiometabolic alterations and a pro-inflammatory status. Our results are suggestive that *ADIPOQ* and *IL6* polymorphisms contribute to cardiometabolic risk in obese individuals.

## Background

Obesity is a multifactorial clinical condition caused by excessive adiposity that is a major contributor to the global epidemic of type 2 diabetes (T2DM), fatty liver disease and cardiovascular disease (CVD) [1,2].

Adipose tissue is a key endocrine organ, which produces several bioactive molecules (adipokines), such as leptin, resistin and adiponectin, with pro- or anti-inflammatory activities [3]. Adipokines are involved in the regulation of appetite and satiety, energy expenditure, endothelial function, hemostasis, blood pressure, adipogenesis and fat distribution, and insulin secretion and sensitivity [4]. Therefore, expansion of adipose tissue changes the secretion of adipokines towards a pro-inflammatory, diabetogenic and atherogenic pattern [3-5].

Adiponectin is an adipokine that is involved in the sensitivity to insulin. Adiponectinemia is markedly decreased in individuals with visceral obesity and states of insulin resistance, such as nonalchoolic fatty liver disease, atherosclerosis, and T2DM [6]. Therefore, adiponectin has been considered as a biomarker for insulin resistance, T2DM, metabolic syndrome and cardiovascular disease.

Adipose tissue also secretes several inflammatory molecules, including interleukin-6 (IL-6) that has both local and systemic effects. It has been suggested that IL-6 plays a role in the induction of the inflammatory process linked to obesity [7].

Common single nucleotide polymorphisms (SNPs) in adiponectin (*ADIPOQ*) and IL-6 (*IL6*) genes have been shown to be related to obesity and adiposity-related phenotypes.

The *ADIPOQ* rs2241766 (45 T > G) and rs1501299 (276G > T, c.214 + 62G > T) SNPs have been associated with variability in plasma adiponectin levels [8,9]. Moreover, these variants have been also related to increased body weight, adiposity, metabolic alterations, insulin resistance, and risk for T2DM and coronary artery disease (CAD), in studies from different ethnic populations [10-15].

A common SNP located in the promoter region of *IL6,* known as -174G > C (rs1800795, c.-237C > G) variant was associated with increased risk for overwheight and obesity-related metabolic disorders, especially insulin resistance in various group populations [16-19]. This *IL6* variant was suggested to play a role in the regulation of body mass through their influence on metabolism and energetic homeostasis [20].

We have investigated the relationship between *ADIPOQ* 45 T > G and *IL6* -174G > C variants and obesity and metabolic markers in a sample of our population.

## Subjects and methods

### Study subjects

Two-hundred-forty-nine individuals, aged 30 to 68 yr, were randomly selected at the University Hospital of University of Sao Paulo, Sao Paulo, Brazil. They were informed about the study protocol and those who agreed to participate as volunteers have signed the informed consent. The study protocol was approved by the Ethics Committees of the School of Pharmaceutical Sciences and University Hospital of the University of Sao Paulo (Sao Paulo, Brazil).

Individuals with thyroid, adrenal or gonad dysfunction or other type of secondary obesity, as well as liver, kidney or gastrointestinal disease, and pregnant women or under treatment of oral contraceptives were not included in the study.

All participants answered a questionnaire to collect personal information during an interview. Each individual declared his ethnic group and provide information about medication use, physical activity, alcohol consumption and cigarette smoking.

Anthropometric measurements, such as body mass index (BMI), waist circumference, waist-to-hip ratio (WHR) were taken from each participant. Individuals were grouped as normoweight (BMI ≤ 24.9 kg/m^2^), overweight (BMI 25.0-29.9 kg/m^2^) and obese (BMI ≥30 kg/m^2^). Body fat and basal metabolic rate (BMR) were measured using a 310E impedanciometer (RJL Systems, Inc, USA).

Systolic/diastolic blood pressure was measured in supine position after resting for 30 min by a trained physician using a mercury column sphygmomanometer. Subjects with systolic/diastolic blood pressure over to 140/90 mmHg or were under anti-hypertensive therapy were considered hypertensive.

Participants with fasting glycemia over 125 mg/dL (American Diabetes Association), or taking a glucose-lowering drug were classified as type 2 diabetics. Current tobacco smoking was considered as a daily intake of one or more cigarette. Alcohol consumption was considered an intake of any dose of beer, wine and/or distilled spirits according to World Health Organization recommendation. Physical exercise practice was considered the practice of sports, for example walking, running or swimming, for at least 2 h per week.

### Laboratory tests

Blood samples were drawn for genomic DNA extraction and measurements of metabolic and inflammatory markers after fasting for 12 h. Serum glucose, total cholesterol, triglycerides and high density lipoprotein (HDL) cholesterol were measured by enzyme-colorimetric methods and apolipoprotein (apo) AI and B were determined by immunoturbidimetry (Siemens Healthcare Diagnosis Inc., Tarrytown, NY, USA). Values of very-low (VLDL) and low density lipoprotein (LDL) cholesterol were calculated.

Fibrinogen was determined using the Fibre Kit-Test Diagnostica Stago Inc (Bayer/STACompact-Stago, Germany). High sensitive C reactive protein (hsCRP) and insulin were determined by immunonephelometry and chemiluminescence, respectively (Siemens Healthcare Diagnosis Inc., Tarrytown, NY, USA). HbA1c was measured in EDTA-anticoagulated blood by affinity chromatography (HPLC) using the D10 Hemoglobin Testing System (Biorad ®, San Francisco, USA). The homeostasis model assessment (HOMA) was used for evaluation of the beta-cell secretory function (HOMA-β) and insulin resistance (HOMA-IR).

Leptin, adiponectin, tumor necrosis factor alpha (TNFα), IL-6, interleukin 1beta (IL-1β), and plasminogen activator inhibitor 1 (PAI-1) in serum were measured by Milliplex technology Luminex Map ® 100/200 (Gen-Probe, Austin, TX, USA). Soluble leptin receptor (sLEPR) was measured by ELISA using kit from ALEXIS Biochemicals Axxora, LLC (San Diego, CA, USA).

### Genetic analysis

Genomic DNA was extracted from peripheral blood leukocytes using the method of affinity chromatography system using QIAGEN spin-column kits and robotic workstation to purify DNA QIAcube (Qiagen Biotechnology Brasil Ltda, Sao Paulo, Brazil).

The *ADIPOQ* 45 T > G (rs2241766) SNP was genotyped by TaqMan real time PCR using the pre-designed assay ID c__26426077_10 for allelic discrimination, containing specif probes for each allele marked with VIC and FAM fluorescent dyes (Applied Biosystems, Foster City, USA). The real time PCR was performed in a ABI PRISM 7500 FAST (Applied Biosystems, Foster City, CA, USA) using the following program: a start cycle of 2 min at 50°C, one cycle of 10 min at 95°C, and 40 cycles of 15 s at 95°C and 1 min at 60°C. The fluorescence signals were analyzed using the program Sequence Detection Software V 1.2.3 (Applied Biosystems, Foster City, USA) that generates clusters of signal amplification which allow the identification of each genotype. Samples with each genotype were analyzed together as an internal control.

The *IL6* -174G > C (rs1800795) SNP was detected by PCR-RFLP. The PCR primers were designed based on the *IL6* sequence [NM_000600] (GenBank, NIH/NCBI) using the Primer Premier® v.5.0 software (PremierBiosoft International, USA), as follows: forward, 5′-TGACTTCAGCTTTACTCTTTGT-3′; and reverse, 5′-CTGATTGGAAACCTTATTAA-3′. The genotypes were identified by an end point PCR assay, using 50 ng DNA, 200 nmol/L primers (IDT, Coralville, IA, USA), 200 μmol/L dNTPs (GE Healthcare, Amersham Biosciences do Brasil, São Paulo, Brazil), 1 U DNA polymerase (BioTools, Madrid, Spain), PCR buffer [75 mM Tris–HCl (pH 9.0), 50 mM KCl, 20 mM (NH_4_) _2_SO_4_, 2 mmol/L MgCl_2_] in 50 μL. PCR assays were carried out in a Mastercycler® (Eppendorf AG, Hamburg, Germany) using the following program: a cycle of 95°C for 1 min, 30 cycles of 95°C for 1 min, 59°C for 1 min and 72°C for 1 min; and a cycle of 72°C for 10 min. The 226pb-amplicon was digested with the endonuclease *Nla* III according to the manufacturer (New England Biolabs Inc., Ipswich, MA, USA). Restriction fragments were identified by 2% agarose gel eletrophoresis.

DNA genotyping results were interpreted by two independent analysts and 20% of DNA samples were randomly re-analyzed.

### Statistical analysis

The results were analyzed using the SigmaStat v. 2.03 (Systat software Inc., San Jose, CA, USA) and Minitab v.15 statistical software (Minitab Inc. State College, PA, USA), assuming significance level of *p* < 0.05. Chi-square test was used to compare categorical variables and the agreement of genotypes frequencies with Hardy-Weinberg equilibrium (HWE) expectations. Continuous variables were compared by *t*-test or Mann–Whitney Rank sum test, as well as ANOVA and Kruskal Wallis with all pairwise multiple comparison using Holm-Sidak and Dunn methods, respectively. Multiple linear regression analysis was used to establish correlations between body mass index and independent variables, considering the covariates: age, gender, ethnics, hypertension, tobacco smoking, alcohol consumption, physical exercise practice, T2DM and CAD. Nominal logistic regression analysis was performed to evaluate the influence of clinical, biochemical and genetic parameters on the risk for overweight and obesity, using the normoweith as reference group.

## Results

Table 1 shows clinical, anthropometrics and laboratory data of the individuals enrolled in this study. Female was the predominant gender in this sample, with higher proportion in normoweight than in overweight and obese groups (p = 0.035). On the other hand, menopause, hypertension, T2DM was less frequent in normoweight individuals (p < 0.05), while obese group was less prone to practice of physical activity (p = 0.003). Mean age, ethnics, family history of CAD, tobacco smoking and alcohol consumption had similar values among the studied groups (p > 0.05). As expected, measurements of BMI, waist circumference, WHR, body fat content and BMR were higher in the obese group and lower in normoweight group (p < 0.001). The leptinemia was higher and sLEPR levels were lower in obese compared with those from normoweight individuals (p < 0.05), while no differences were found in adiponectin plasma concentration among the groups (p > 0.05).

**Table 1:** **Clinical, anthropometrics, laboratory and polymorphism data of the studied group**

**Variable**	**Normoweight (51)**	**Overweight (53)**	**Obese (145)**	***P-value***
Age, years	46.6. ± 8.3	47.9 ± 6.9	48.7 ± 9.2	*0.323*
Ethnics [White], %	78.4 (40)	69.8 (37)	71.0(103)	*0.539*
Woman, %	92.2 (47)	79.2 (42)	75.2 (109)	***0.035***
Menopause,%	10.6 (5)	26.2 (11)	33.9 (37)	***0.011***
Hypertension, %	13.7 (7)	28.3 (15)	45.5 (66)	***<0.001***
Type 2 diabetes, %	33.3 (17)	41.5 (22)	55.7 (99)	***<0.001***
Family history of CAD	11.7 (6)	20.8 (11)	17.9 (26)	*0.455*
Tobacco smoking,%	17.6 (9)	23.2 (10)	13.1 (19)	*0.527*
Alcohol consumption, %	2.0 (1)	3.8 (2)	3.4 (5)	*0.845*
Physical exercise practice,%	50.9 (26)	50.9 (27)	29.6 (43)	***0.003***
Body mass index, kg/m^2^	22.6 ± 1.7a	27.4 ± 1.4b	35.1 ± 4.2c	***<0.001***
Waist circumference, cm	73.9 ± 8.7a	86.8 ± 9.4b	104.1 ± 11.0c	***<0.001***
Waist- hip ratio	0.78 ± 0.07a	0.84 ± 0.08b	0.90 ± 0.10 c	***<0.001***
Body fat, %	30.9 ± 4.7a	33.9 ± 6.4b	38.5 ± 5.0c	***<0.001***
BMR, kcal	1272 ± 240a	1501 ± 295b	1742 ± 280c	***<0.001***
Leptin, ng/mL	13.6 ± 8.1a	15.7 ± 12.0a,b	22.9 ± 19.2b	***0.012***
sLEPR, ng/mL	23.8 ± 12.4a	19.1 ± 13.5a,b	20.0 ± 24.0b	***0.011***
Adiponectin, μg/mL	24.6 ± 23.2	17.7 ± 23.0	27.2 ± 26.4	*0.070*
Glucose, mg/dL	92 ± 9a	102 ± 26a,b	106 ± 29b	***<0.001***
HbA1c, %	5.5 ± 0.4a	6.0 ± 1.3a,b	6.0 ± 1.4b	***0.005***
Insulin, mU/L	6.6 ± 4.3a	9.7 ± 6.1b	21.3 ± 12.2b	***<0.001***
HOMA-β	23.0 ± 18.2a	33.3 ± 24.5b	68.7 ± 37.2b	***<0.001***
HOMA-IR	1.5 ± 1.0a	2.4 ± 1.5b	5.9 ± 4.8b	***<0.001***
Total cholesterol, mg/dL	194 ± 36	203 ± 37	209 ± 40	*0.077*
HDL ccholesterol, mg/dL	65 ± 19a	55 ± 17b	51 ± 12b	***<0.001***
LDL cholesterol, mg/dL	113 ± 32a	125 ± 32a,b	128 ± 32b	***0.016***
VLDL cholesterol, mg/dL	16 ± 8a	23 ± 10b	30 ± 18c	***<0.001***
Triglycerids, mg/dL	79 ± 38a	114 ± 53b	150 ± 88c	***<0.001***
Apolipoprotein AI, mg/dL	160 ± 40a	152 ± 36a,b	146 ± 31b	***0.009***
Apolipoprotein B, mg/dL	89 ± 28a	109 ± 38b	101 ± 28b	***0.004***
Fibrinogen, mg/dL	341 (304–374)a	350 (319–409)a,b	374 (328–446)b	***<0.001***
PAI-1, ng/mL	62.5 (44.7-76.1)a	63.6 (51.0-95.4)a	96.1(60.0-150.6)b	***<0.001***
IL-6, pg/mL	0.08 (0.07-0.09)a	0.09 (0.08-0.55)a,b	0.56 (0.28-1.6)c	***<0.001***
IL-1β, pg/dL	5.0 (4.0-5.6)a	5.9 (4.0-6.1)a,b	18.0 (15.0-51.5)c	***<0.001***
hsCRP, mg/L	0.14 (0.05-0.58)a	0.49 (0.08-1.17)a,b	1.72 (0.26-5.69)c	***<0.001***
TNFα, pg/mL	0.56 (0.09-1.25)a	0.71 (0.32-2.85)a,b	3.17 (1.45-6.05)c	***<0.001***
Minor Allele Frequency, %				
*ADIPOQ* 45 T > G (rs2241766)	19.6	22.6	21.7	*0.858*
*IL6* -174G > C (rs1800795)	26.4	37.7	31.0	*0.209*

Number of individuals is in parenthesis. Results are shown as mean ± SD or median (interquartile range) and compared by Anova (Multiple comparisons by Holm-Sidak method) or Kruskal Wallis (Dunn’s Method). Different letters indicate differences between mean values. Categorical variables were compared by chi-square. BMR: basal metabolic rate; CAD: coronary artery disease; HDL: high-density lipoprotein; hsCRP: high sensitive C reactive protein; LDL: low-density lipoprotein; PAI-1: plasminogen activator inhibitor-1; IL-6: interleukin 6; IL-1β: interleukin 1beta; sLEPR: soluble leptin receptor; TNFα: tumor necrosis factor alpha; VLDL: very low-density lipoprotein.

Obese subjects had higher levels of glucose, HbA1c, HOMA-β and HOMA-IR than normoweight group (p < 0.05). Obeses also had a more atherogenic profile with higher levels of LDL and VLDL cholesterol, tryglicerides and apoB and lower HDL cholesterol and apoAI compared with the normoweight group (p < 0.05). The values of fibrinogen, PAI-1, IL-6, IL-1β, TNFα and hsCRP were also higher in obese than in normoweight subjects (p < 0.05) (Table 1).

Results from multiple linear regression analysis confirmed that waist circumference, WHR and body fat were positively correlated with BMI values (p < 0.001) (Table 2). In addition, an increment of one unit in the plasma concentration of leptin, IL-1β, hsCRP, TNFα, PAI-1 and fibrinogen were related respectively with an increase of 0.12, 0.11, 0.31, 0.35, 0.12, 0.02 Kg/m^2^ of BMI in the study group (p < 0.05).

**Table 2:** **Anthropometric, biochemical and genetic variables as predictors for body mass index values: multiple linear regression analysis**

**Independent variables**	**B**	**SE**	***P-value***
*Anthopometrics*			
Waist circumference, cm	0.361	0.017	***<0.001***
Body fat, %	0.840	0.055	***<0.001***
Waist-hip ratio	21.317	4.397	***<0.001***
*Biochemical parameters*			
Fibrinogen, mg/dL	0.021	0.004	***<0.001***
PAI-1, pg/mL	0.121	0.038	***0.002***
hsCRP, mg/L	0.31	0.07	***<0.001***
IL-6, pg/mL	−0.006	0.118	*0.962*
IL-1β, pg/dL	0.110	0.018	***<0.001***
TNFα, pg/mL	0.348	0.130	***0.008***
Adiponectin, μg/mL	0.0023	0.0170	*0.890*
Leptin, ng/mL	0.120	0.024	***<0.001***
sLEPR, ng/mL	0.0007	0.0232	*0.975*
*Polymorphisms*			
*ADIPOQ* 45 T > G (ref TT)	0.259	0.773	*0.738*
*IL6* -174G > C (ref GG)	0.099	0.740	*0.893*

Regression coefficients (B) and standard errors (SE) are expressed in kg/m^2^. Age, gender, ethnics, hypertension, tobacco smoking, alcohol consumption, physical exercise practice, T2DM and CAD were used as covariates. Polymorphisms were introduced as dummy variables for absence or presence of the rare allele.

Univariate logistic regression analysis was carried out to evaluate the influence of clinical, biochemical and genetic variables as risk factors for overweight and obesity. As shown in Table 3, waist circumference, body fat and dyslipidemia increased the risk for overweight and obesity in this sample (p < 0.05). In addition, hypertension and T2DM increase more than four times the risk for obesity (p < 0.001), while female gender and physical activity reduce this risk (p < 0.05). Increased plasma levels of both fibrinogen and IL-1β were associated with high risk for overweight and obesity (p < 0.05) (Table 3). Moreover, increased leptin, hsCRP and TNFα concentrations were also risk factors for obesity (p < 0.05).

**Table 3:** **Influence of clinical, biochemical and genetic variables on the risk for overweight and obesity: Univariate logistic regression analysis**

**Independent variables**	***Risk for overweight***			***Risk for Obesity***		
	**OR**	**95% CI**	***P-value***	**OR**	**95% CI**	***P-value***
*Clinical and anthropometric variables*						
Age, years	1.02	0.97 – 1.06	*0.453*	1.03	0.99 – 1.07	*0.145*
Gender [ref: male]	0.37	0.11 – 1.25	*0.110*	0.25	0.08 – 0.73	***0.011***
Ethnics [ref: white]	0.64	0.26 – 1.55	*0.318*	0.67	0.31 – 1.43	*0.297*
Menopause	1.13	0.50 – 2.52	*0.774*	0.54	0.27 – 1.08	*0.081*
Hypertension	2.48	0.92 – 6.72	*0.074*	5.32	2.25 – 12.60	***<0.001***
T2DM	1.42	0.64 – 3.15	*0.390*	4.40	2.23 – 8.69	***<0.001***
Dyslipidemia	3.30	1.48 – 7.38	***0.004***	5.02	2.53 – 9.97	***<0.001***
Family history of CAD						
Tobacco smoking	1.09	0.40 – 2.94	*0.872*	0.71	0.30 – 1.69	*0.437*
Alcohol consumption	1.96	0.17 – 22.32	*0.587*	1.80	0.21 -15.77	*0.596*
Physical exercise practice	0.93	0.43 – 2.00	*0.845*	0.41	0.21 – 0.79	***0.008***
Waist circumference, cm	1.21	1.12 – 1.30	***<0.001***	1.42	1.30 – 1.55	***<0.001***
Body fat, %	1.13	1.04 – 1.23	***0.004***	1.34	1.23 – 1.46	***<0.001***
*Biochemical parameters*						
Leptin, ng/mL	1.02	0.98 – 1.06	*0.434*	1.05	1.01 – 1.08	***0.006***
sLEPR, ng/mL	0.99	0.90 – 1.01	*0.397*	0.99	0.97 – 1.01	*0.421*
Adiponectin, μg/mL	0.99	0.97–- 1.01	*0.155*	1.00	0.99 – 1.02	*0.555*
Fibrinogen, mg/dL	1.01	1.00 – 1.01	***0.037***	1.01	1.00 – 1.01	***<0.001***
PAI-1, pg/mL	1.03	0.98 – 1.08	*0.204*	1.04	1.00 – 1.08	*0.057*
IL-6, pg/mL	1.23	0.91 – 1.67	*0.183*	1.24	0.92 – 1.66	*0.154*
IL-1β, pg/dL	1.08	1.01 – 1.16	***0.029***	1.14	1.06 – 1.22	***<0.001***
hsCRP, mg/L	1.24	0.87 – 1.76	*0.232*	1.75	1.28 – 2.3	***<0.001***
TNFα, pg/mL	1.18	0.96 – 1.45	*0.123*	1.35	1.13 – 1.62	***0.001***
*Polymorphisms*						
*ADIPOQ* 45 T > G (ref TT)	1.03	0.46 - 2.32	*0.946*	1.06	0.54 - 2.09	*0.858*
*IL6* -174G > C (ref GG)	1.50	0.68 - 3.30	*0.317*	0.95	0.50 - 1.81	*0.884*

Results obtained from a univariate nominal regression analysis using the normoweight subjects as reference group. OR, odds ratio; CI, confidence interval. Polymorphisms were introduced as dummy variables for absence or presence of the rare allele.

**Figure 1:**
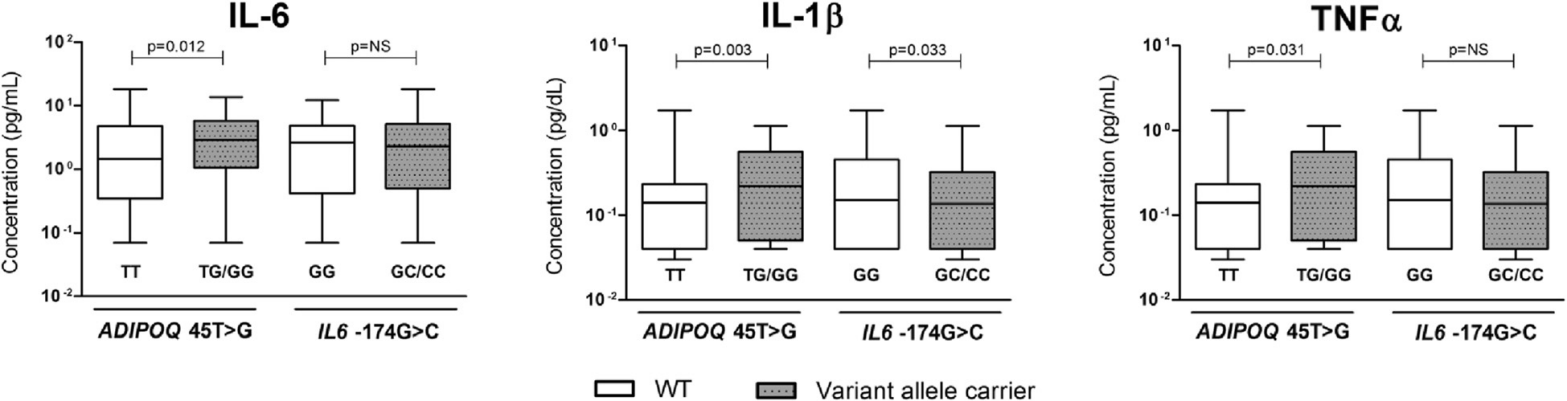
Influence of *ADIPOQ* 45 T > G and *IL6* -174G > C polymorphisms on plasma inflammatory biomarkers. Box plots represent plasma concentration of IL-6, IL-1β and TNF-α according to *ADIPOQ* 45 T > G and *IL6* -174G > C genotypes. Values of wild type genotypes (WT) were compared with variant allele carriers for each polymorphism using the Mann–Whitney Rank sum test. *p*-values are indicated in the figure for each comparison. NS: no significant.

*ADIPOQ* 45 T > G and *IL6* -174G > C SNPs had genotype distributions as expected from HWE in this sample (Data not shown). Minor allele frequencies of these variants were similar between obese, overweight and normoweight groups (p > 0.05) (Table 1). These variants did not show significant association with neither BMI (Table 2) nor risk for overwheight and obesity (table 3) in this sample. Interestingly, individuals carrying *ADIPOQ* 45G allele (TG + GG genotype) had higher IL-6, IL-1β and TNFα plasma levels than TT genotype carriers (p < 0.05) (Figure 1, Table 4). While *IL6* -174GG genotype was associated with higher levels of IL-1β (p = 0.033) (Figure 1, Table 5).

**Table 4:** **Relationship of** ***ADIPOQ*** **45 T > G with anthropometric, metabolic and inflammatory variables**

**Variable**	**Genotypes**		
	**TT (163)**	**TG + GG (86)**	***P-value***
Body mass index, kg/m^2^	30.9 ± 6.2	31.1 ± 6.2b	*0.818*
Waist circumference, cm	94.4 ± 16.4	94.1 ± 15.3	*0.881*
Waist- hip ratio	0.86 ± 0.10	0.87 ± 0.10	*0.249*
Body fat, %	35.8 ± 6.0	36.3 ± 6.5	*0.527*
BMR, kcal	1611 ± 336	1530 ± 328	*0.190*
Leptin, ng/mL	20.5 ± 17.6	18.8 ± 15.8	*0.666*
sLEPR, ng/mL	21.0 ± 21.9	19.6 ± 18.6	*0.614*
Adiponectin, μg/mL	25.7 ± 25.4	21.5 ± 24.0	*0.442*
Glucose, mg/dL	101 ± 25	104 ± 27	*0.464*
HbA1c, %	5.9 ± 1.2	5.9 ± 1.3	*0.476*
Insulin, mU/L	15.3 ± 1.6	17.6 ± 12.7	*0.181*
HOMA-β	50.7 ± 38.6	56.0 ± 35.3	*0.205*
HOMA-IR	4.1 ± 4.0	5.0 ± 4.8	*0.153*
Total cholesterol, mg/dL	205 ± 36	204 ± 43	*0.663*
HDL ccholesterol, mg/dL	55 ± 15	53 ± 16	*0.205*
LDL cholesterol, mg/dL	125 ± 32	124 ± 34	*0.816*
VLDL cholesterol, mg/dL	25 ± 14	27 ± 19	*0.475*
Triglycerides, mg/dL	124 ± 70	135 ± 93	*0.475*
Apolipoprotein AI, mg/dL	151 ± 35	148 ± 33	*0.557*
Apolipoprotein B, mg/dL	100 ± 27	100 ± 38	*0.469*
Fibrinogen, mg/dL	361 (319–423)	353 (316–399)	*0.327*
PAI-1, pg/mL	74.5 (55.4-138.8)	81.9 (58.2-137.7)	*0.713*
IL-6, pg/mL	0.28 (0.08-0.64)	0.54 (0.28-1.4)	***0.012***
IL-1β, pg/dL	14 (4.0-23.0)	22 (4.6-55.7)	***0.003***
hsCRP, mg/L	0.54 (0.1-3.1)	0.56 (0.14-3.0)	*0.980*
TNFα, pg/mL	1.45 (0.34-4.81)	2.9 (1.06-5.84)	***0.031***

Number of individuals is in parenthesis. Results are shown as mean ± SD or median (interquartile range) and compared by Mann–Whitney Rank sum test. BMR: basal metabolic rate; CAD: coronary artery disease; HDL: high-density lipoprotein; LDL: low-density lipoprotein; hsCRP: high sensitive C reactive protein; IL-6: interleukin 6; IL-1β: interleukin 1beta; PAI-1: plasminogen activator inhibitor-1; sLEPR: soluble leptin receptor; TNFα: tumor necrosis factor alpha; VLDL: very low-density lipoprotein.

**Table 5:** **Relationship of** ***IL6*** **-174G > C with anthropometric, metabolic and inflammatory variables**

**Variable**	**Genotypes**		
	**GG (112)**	**GC + CC (137)**	***P-value***
Body mass index, kg/m^2^	31.2 ± 6.1	30.7 ± 6.3	*0.528*
Waist circumference, cm	96.4 ± 16.1	92.6 ± 15.7	*0.067*
Waist- hip ratio	0.87 ± 0.10	0.85 ± 0.10	*0.164*
Body fat, %	35.5 ± 6.0	36.4 ± 6.2	*0.246*
BMR, kcal	1644 ± 373	1543 ± 298	*0.057*
Leptin, ng/mL	21.8 ± 18.6	18.6 ± 15.6	*0.394*
sLEPR, ng/mL	22.4 ± 23.3	19.2 ± 18.8	*0.615*
Adiponectin, μg/mL	24.0 ± 24.0	24.5 ± 25.9	*0.755*
Glucose, mg/dL	103 ± 31	102 ± 21	*0.072*
HbA1c, %	6.0 ± 1.4	5.9 ± 1.1	*0.876*
Insulin, mU/L	17.7 ± 13.6	14.3 ± 9.8	*0.237*
HOMA-β	57.0 ± 40.3	47.4 ± 34.4	*0.148*
HOMA-IR	4.9 ± 5.3	3.7 ± 2.7	*0.379*
Total cholesterol, mg/dL	205 ± 42	204 ± 36	*0.755*
HDL ccholesterol, mg/dL	56 ± 19	54 ± 12	*0.823*
LDL cholesterol, mg/dL	122 ± 34	127 ± 31	*0.241*
VLDL cholesterol, mg/dL	26 ± 19	25 ± 13	*0.759*
Triglycerides, mg/dL	132 ± 94	124 ± 64	*0.759*
Apolipoprotein AI, mg/dL	152 ± 35	148 ± 34	*0.753*
Apolipoprotein B, mg/dL	101 ± 27	100 ± 34	*0.462*
Fibrinogen, mg/dL	355 (317–407)	365 (319–420)	*0.325*
PAI-1, pg/mL	82.7 (57.5-133.5)	75.2 (56.3-145.9)	*0.991*
IL-6, pg/mL	0.48 (0.08-0.64)	0.28 (0.08-0.96)	*0.288*
IL-1β, pg/dL	15.0 (4.0-45.0)	13.5 (4.0-32.0)	***0.033***
hsCRP, mg/L	0.57 (0.13-3.2)	0.52 (0.10-2.94)	*0.808*
TNFα, pg/mL	2.62 (0.41-4.92)	2.29 (0.49-5.18)	*0.666*

Number of individuals is in parenthesis. Results are shown as mean ± SD or median (interquartile range) and compared by Mann–Whitney Rank sum test. BMR: basal metabolic rate; CAD: coronary artery disease; HDL: high-density lipoprotein; LDL: low-density lipoprotein; hsCRP: high sensitive C reactive protein; IL-6: interleukin 6; IL-1β: interleukin 1beta; PAI-1: plasminogen activator inhibitor-1; sLEPR: soluble leptin receptor; TNFα: tumor necrosis factor alpha; VLDL: very low-density lipoprotein.

## Discussion

Results from this study demonstrate an association of obesity with hypertension, T2DM, insulin resistance and an atherogenic lipid profile, confirming that overweight and obese individuals are more susceptible to metabolic dysfunction and atherosclerosis, which are known risk factors for CVD [21].

The hyperleptinemia and reduced sLEPR found in overweight and obese patients indicate a status of leptin resistance, which is probably caused by dysregulation of the negative feedback, as a classic mechanism of hormone resistance [22]. It is well known that hyperleptinemia triggers a chronic overstimulation of the leptin receptor and activation of negative feedback pathways that block further leptin signaling, leading to leptin resistance (22). The hyperleptinemia has been attributed to a deficiency in leptin transport through the blood–brain barrier, as well as, to the presence of variants in *LEPR*, which alters funcionatily or even expression of the receptor reducing its circulating levels, as found in obese subjects.

It has been suggested that resistance to leptin produce metabolic and inflammatory alterations in several tissues and organs, including the liver, spleen and heart, therefore leptin resistance contributes to the risk for obesity-related comorbidities [23].

In this work, obesity was associated with hyperglycemia, insulin resistance and dyslipidemia. Obese patients have reduced supply of glucose in adipocytes that leads to a decreased intracellular lipolysis and increased release of non-esterified fatty acids, which results in insulin resistance, dysglycemia and dyslipidemia [24]. These metabolic alterations increase the risk for CVD in obese subjects.

We could not find a direct relationship of the *ADIPOQ* 45 T > G polymorphism with obesity or variability in body fat mass, waist circumference and BMI values. Similarly, this variant was not associated with BMI in other groups such as Mexican-Mestizos [25], Tunisian volunteers [26], Saudi Arabians [27] and Chinese [28]. This lack of association was also found in groups of women [29] or men [10,11] suggesting that is independent on gender.

The *ADIPOQ* 45 T > G variant has been shown to be associated with variability in adiponectin levels in several studies including in overweight Finish subjects with impaired glucose tolerance [12], diabetic and non-diabetic Brazilian patients with high cardiovascular risk [15], Arab patients with acute coronary syndrome [30]. However, this relationship was not found in our study and in other population samples [25-29], including patients with coronary artery disease [31,32].

We did not find an association between *IL6* -174G > C SNP and obesity or variability in BMI. A large study with two independent cohorts has indicated that *IL6* variants are significantly associated with adiposity, but the contribution of the SNP -174G > C seems to be less likely [33]. Lack of relationship of this variant and obesity was also found in children [34], adolescents [35] and diabetic adults [36] suggesting that it is not a major contributor to obesity risk.

In this study, BMI was positively correlated with circulating levels of pro-inflammatory such as fibrinogen, PAI-1, hsCRP, IL-1β, and IL-6 and TNFα. Moreover, altered levels in most of these pro-inflammatory markers were associated with increased risk for obesity. These results confirm previous studies, which suggested that the expansion of the adipose tissue favours the development of a pro-inflammatory, diabetogenic and atherogenic status [3,4].

We also found that variability in TNFα, IL-1β and IL-6 circulating levels was associated with *ADIPOQ* 45 T > G SNP, suggesting a role of this variant in regulation of the pro-inflammatory status in obese subjects. Guzman-Ornelas et al. have also found an association of *ADIPOQ* 45 T > G SNP with a pattern of fat distribution and correlations with inflammation markers, but not directely related to the genotypes [26].

*IL6* -174G > C SNP did not influence the IL-6 circulating levels as it has been shown in a study with Spanish adolescents that demonstrated an association between fat mass and cardiovascular risk factor [37]. Conversely, in Italian Caucasian females, increased plasma levels of IL-6 were found in obese individuals carrying GG genotype suggesting that fat mass is a major determinant of an increase in IL-6 production and insulin resistance [38].

IL-1β levels were influenced by *IL6* -174G > C SNP in this sample, with high levels found in individuals carrying GG genotype compared to C allele (GC + CC genotypes) carriers. In a similar way, Mendoza-Carrera et al. found an association between C allele and low hsCRP levels in the overweight adolescents [35]. Moreover Ramírez-López et al. reported a relationship between GCG/GCG haplotype from *IL6* -597G > A, −572G > C and -174G > C SNPs and high hsCRP [39]. These results are suggestive of a protective effect of the *IL6* -174G > C variant on inflammatory status of obese individuals.

Even though the results from this study are interesting the lack of association between *ADIPOQ* variant and obesity and adiponectin levels may be influenced by the heterogeneity and size of our sample population and also environmental factors.

## Conclusion

The results from this study confirm that obesity is associated with cardiometabolic alterations, leptin resistance and a pro-inflammatory status. Our results are suggestive that *ADIPOQ* and *IL6* polymorphisms contribute to cardiometabolic risk in obese individuals; however, more studies using higher sample sizes are needed to confirm the associations observed in this work.

## Abbreviations

ADIPOQ: Adiponectin
BMI: Body mass index
BMR: Basal metabolic rate
CAD: Coronary artery disease
CVD: Cardiovascular disease
hsCRP: High sensitive C reactive protein
IL-1β: Interleukin 1 beta
IL-6: Interleukin 6
LEPR: Leptin receptor
PAI-1: Plasminogen activator inhibitor-1
sLEPR: Soluble leptin receptor
SNP: Single nucleotide polymorphism
T2DM: Type 2 diabetes mellitus
TNFα: Tumor necrosis factor alpha
WHR: Waist-to-hip ratio
